# Insulin in Central Nervous System: More than Just a Peripheral Hormone

**DOI:** 10.1155/2012/384017

**Published:** 2012-02-21

**Authors:** Ana I. Duarte, Paula I. Moreira, Catarina R. Oliveira

**Affiliations:** ^1^CNC, Center for Neuroscience and Cell Biology, University of Coimbra, 3004-517 Coimbra, Portugal; ^2^Institute of Physiology, Faculty of Medicine, University of Coimbra, 3000-354 Coimbra, Portugal; ^3^Institute of Biochemistry, Faculty of Medicine, University of Coimbra, 3000-354 Coimbra, Portugal

## Abstract

Insulin signaling in central nervous system (CNS) has emerged as a novel field of research since decreased brain insulin levels and/or signaling were associated to impaired learning, memory, and age-related neurodegenerative diseases. Thus, besides its well-known role in longevity, insulin may constitute a promising therapy against diabetes- and age-related neurodegenerative disorders. More interestingly, insulin has been also faced as the potential missing link between diabetes and aging in CNS, with Alzheimer's disease (AD) considered as the “brain-type diabetes.” In fact, brain insulin has been shown to regulate both peripheral and central glucose metabolism, neurotransmission, learning, and memory and to be neuroprotective. And a future challenge will be to unravel the complex interactions between aging and diabetes, which, we believe, will allow the development of efficient preventive and therapeutic strategies to overcome age-related diseases and to prolong human “healthy” longevity. Herewith, we aim to integrate the metabolic, neuromodulatory, and neuroprotective roles of insulin in two age-related pathologies: diabetes and AD, both in terms of intracellular signaling and potential therapeutic approach.

## 1. Introduction

Almost all cell types are responsive to insulin. However, liver, muscle, and adipose tissue are the most sensitive to the hormone [1], rendering it the most important anabolic hormone identified to date. In vertebrates, this peptide belongs to a superfamily of structurally related proteins, the insulin-related family of substances, that includes insulin-like growth factors-1 (IGF-1) and -2 (IGF-2) and relaxin [2].

Until three decades ago, insulin was considered only as a peripheral hormone, unable to cross the blood-brain barrier (BBB) and to affect the central nervous system (CNS) [3–5]. However, this idea was challenged after the detection of immunoreactive insulin in dog cerebrospinal fluid (CSF) [6]. Further studies provided clear evidence that insulin occurs in brain, where it may reach high levels [7, 8], exerting long-term trophic effects on CNS neurons [2]. Although the in vivo brain insulin levels remain controversial, they appear to be 10- to 100-fold higher than in plasma and to change during brain development, with the highest values in late fetal and early neonatal rabbit brain (about 80–90 and 195 ng/g, respectively), that decrease in the adult brain (about 32 ng/g) [7]. 

Insulin present in adult CNS is primarily derived from pancreatic *β*-cells and is transported by CSF into the brain [3–5, 9, 10]. This insulin crosses BBB mostly via a carrier-mediated, saturable, regulatable, and temperature-sensitive active process [4, 5, 9, 10] that is limited by the barrier system formed by the tight junctions between endothelial cells [2]. Thus, it is not surprising that an acute increase in peripheral insulin levels leads to higher CSF insulin, whilst chronic peripheral hyperinsulinemia (as occurs in insulin resistance) downregulates insulin receptors (IR) at BBB, impairing insulin transport into the brain [11]. Moreover, an increase in circulating insulin has been shown to rapidly affect brain activity (independently from its systemic effects), as occurs in healthy individuals submitted to a hyperinsulinemic-euglycemic clamp [12]. Alternatively, peripheral insulin can access CNS directly through the area postrema, a circumventricular region with a “leaky” BBB that allows free diffusion of plasma solubles directly into this area [13, 14]. 

*De novo *insulin synthesis in brain has been proposed as an alternative source of insulin in CNS. This hypothesis has been supported by the detection of preproinsulin I and II mRNA in rat fetal brain and cultured neurons and also by insulin immunoreactivity in neuronal endoplasmic reticulum, Golgi apparatus, cytoplasm, axon, dendrites, and synapses [15–21]. Additionally, high levels of insulin in brain extracts [22], its presence in immature nerve cell bodies [7, 18], the rapid transport of peripherally injected insulin into the CSF [12], and the fact that less than 1% of the hormone crosses the BBB in dogs and rodents [23] further support the idea that insulin can be synthesized in brain. However, the unequivocal evidence supporting this hypothesis is that insulin can be synthesized in cultured rat brain neurons and released upon K^+^- and Ca^2+^-induced membrane depolarization [24]. More specifically, its synthesis seems to occur in pyramidal neurons (e.g., from hippocampus, prefrontal cortex, enthorhinal cortex, and olfactory bulb), but not in glial cells [25]. Thus, it is not surprising that insulin is highly enriched in brain cortex, olfactory bulb, hippocampus, hypothalamus, and amygdala [26]. Similarly to insulin, insulin-growth factor-1 (IGF-1) occurs also in rodent and human brain and can cross the BBB [11, 27, 28]. 

Taken this together, the next obvious question regards insulin and IR physiological role(s) in brain. Herewith, we will first analyse brain IR-mediated signaling pathways and the pivotal role of insulin in brain (and peripheral) glucose metabolism, synaptic transmission, memory/learning, and neuroprotection. Then, we will focus preferentially on the protective effect of insulin against diabetes and its long-term complications in CNS, aging/longevity, and age-related neurodegenerative disorders (especially Alzheimer's disease (AD)). Finally, we will briefly discuss the pros and cons of the potential therapeutic interest of insulin in diabetes- and age-related neurodegeneration. In fact, from the evidences presented herein, insulin appears to be a naturally occurring hormone essential for normal CNS function.

## 2. Neuronal IR/IGF-1R-Mediated Signaling Pathways

### 2.1. Brain IR/IGF-1R Localization

Once in brain, insulin rapidly binds to IR, which are highly abundant (but selectively distributed) throughout CNS [15, 20, 26, 29], especially in olfactory bulb, hypothalamus, cerebral cortex, cerebellum, hippocampus, and striatum [5, 9, 30]. This differential distribution of insulin and IR in brain suggests that insulin from different sources (peripheral or local) may reach IR from distinct brain regions to initiate neuronal signal transduction [31]. Concerning intracellular localization, IR are highly abundant in neurons (with high protein expression in cell bodies and synapses) and less abundant in glia [32–36]. Similarly, IGF-1 receptors (IGF-1R) were detected throughout neurons and glia [37], particularly in hippocampus, amygdala, parahippocampal gyrus, cerebellum, cerebral cortex, and caudate nucleus, being less abundant in substantia nigra, red nucleus, white matter, and cerebral peduncles [15].

### 2.2. Brain IR/IGF-1R Structure and Signaling Pathways

IR and IGF-1R are tetrameric glycoproteins that belong to the receptor tyrosine (Tyr) kinase superfamily, composed of two *α* (120–135 kDa) and two *β* (95 kDa) subunits [2, 38–40]. Brain IR subunits differ from peripheral ones by the slightly lower molecular weight and by the absence of downregulation after exposure to high insulin levels [20, 41, 42]. Besides IR and IGF-1R, the Tyr kinase superfamily comprises also human insulin receptor-related protein, *Drosophila* homolog of IR and mollusc IR-related protein, suggesting that their features have been highly conserved throughout evolution [2]. Interestingly, two different types of IR have been found in mammalian brain*:* a “peripheral-” like type (with lower density in glia cells) and a neuron-specific type (highly expressed in neurons) [11]. Due to structural and functional homology, insulin and IGF-1 can bind to (and activate) both IR and IGF-1R, with insulin binding to the IR with higher affinity (<1 nM) than IGF-1 (100–500-fold lower affinity), whereas IGF-1R preferentially binds IGF-1 (<1 nM) as compared to insulin (100–500-fold lower affinity) [43]. Once bound to *α* subunits of the neuronal IR or IGF-1R, insulin (or IGF-1) promotes autophosphorylation of the *β* subunit at Tyr residues 1158, 1162, and 1163, triggering its intrinsic Tyr kinase activity [5, 39, 40, 44–46] and phosphorylating insulin receptor substrate (IRS) docking proteins (IRS1-4) at Tyr residues [1]. Then, Src homology-2 (SH2) domain-containing signaling molecules (namely, the p85 regulatory subunit of phospatidylinositol 3-kinase (PI3K)) are recruited and activate the catalytic subunit of PI3K, as well as the growth factor receptor-bound protein 2 (Grb-2) [5, 40, 44, 46]. As a consequence, two major signaling pathways can be activated: the PI3K/Akt/glycogen synthase kinase-3*β* (GSK-3*β*) and the Ras/Raf-1/extracellular signal-regulated kinases (ERK1 and ERK2, ERK1/2) [5, 39, 40, 46].

### 2.3. PI3K/Akt Signaling Cascade

After binding of the p85 SH2 domain to the phosphorylated Tyr of active IRS, PI3K becomes active and p110 inhibition is relieved, allowing translocation of active PI3K to plasma membrane and subsequent formation of PI-3, 4, 5-trisphosphate, and PI-3,4-bisphosphate. Then, these molecules bind to and recruit downstream signaling proteins containing pleckstrin homology domains. Amongst them, the serine (Ser)/threonine (Thr) kinase Akt can be recruited to the plasma membrane [30, 40, 46–48] and phosphorylated by protein kinase 3-phosphoinositide-dependent protein kinase-1 [44, 46, 49]. Once activated, Akt detaches from plasma membrane and translocates into cytosol and nucleus [46], where it phosphorylates target proteins at Ser and Thr residues [4]. These target proteins include the proapoptotic Bad (inactivated when phosphorylated at Ser136), caspase-9 (inhibited by phosphorylation at Ser196), and GSK-3 [4]. Thus, Akt activation inhibits apoptosis [4]. 

Of relevance in this signaling pathway is the GSK-3*β*, a Ser/Thr protein kinase that becomes inactive upon Akt-mediated phosphorylation at the N-terminal Ser9, initiating multiple physiologic effects [39, 50–53]. GSK-3*β* is negatively regulated also by PKC and c-AMP-dependent protein kinase (PKA) (which are also activated by insulin or IGF-1) [54, 55]. Depending on the cellular context, GSK-3*β* can be targeted to different intracellular locations (e.g., cytosol, mitochondria, or nucleus) to easily access its substrates [56]: active GSK-3*β* (Ser9 dephosphorylated) appears mostly in nuclei, mitochondria, and membrane lipid rafts (detergent-resistant plasma membrane microdomains involved in signal transduction), while inactive GSK-3*β* is mostly cytosolic [57]. Besides Ser9 dephosphorylation, GSK-3*β* can be also activated by phosphorylation at Tyr216, and, although this mechanism remains unclear, changes in intracellular Ca^2+^ or Fyn (a member of the Src Tyr kinase family) appear to be involved [58, 59]. The inhibition of GSK3*β*, occurring after PI3K inhibition, has been described to prevent apoptosis, while overexpression of a constitutively active GSK-3*β* resulted in PC12 cell death [60–62]. Interestingly, Lin et al. [63] described that maintenance of phospho-GSK-3*β* levels, after transient activation of PI3K/Akt signalling, prevented apoptosis induced by hydrogen peroxide (H_2_O_2_) in neural stem cells. Moreover, we previously showed that insulin prevented neuronal IR/IGF-1R inactivation upon oxidative stress and, by inhibiting GSK-3*β* (via PI-3K/Akt activation), insulin stimulated the synthesis of proteins involved in neuronal antioxidant defence, glucose metabolism, and antiapoptotic mechanisms [64]. Although the mechanisms underlying GSK-3*β*-induced apoptosis remain unclear, decreased CREB-mediated expression of Bcl-2 [65, 66], mitochondrial cytochrome c release and caspase activation (either via the intrinsic pathway or via translocation of the apoptosis-inducing factor, AIF) [67–70], phosphorylation and downregulation of heat shock proteins (e.g., heat shock factor-1) may be involved [67]. 

Besides GSK-3*β*, IGF-1-induced activation of Akt also phosphorylates and inhibits FoxO3, which is retained in cytoplasm by the 14-3-3 protein. As a result, nuclear translocation of FoxO3 is blocked, as well as its subsequent deleterious targeting of nuclear genes, thus promoting hippocampal and cerebellar granule neuronal survival [49, 71, 72]. This FoxO3 inhibition was also shown to impair Bim transcription in cerebellar granular neurons, contributing to the antiapoptotic effect of IGF-1 [72, 73]. Akt may also phosphorylate nuclear factor-*κ*B (NF-*κ*B) either directly [74, 75] or via IKK-induced inhibitory protein of *κ*B (I*κ*B) phosphorylation at Ser32 (with the concomitant disintegration of the p65-NF-*κ*B/I*κ*B complex, translocation of p65-NF-*κ*B into the nucleus, and its heterodimerization with other NF-*κ*B members for transcriptional regulation) [76, 77]. Importantly, NF-*κ*B activation has been shown to increase Cu/Zn -superoxide dismutase (SOD) expression and MnSOD levels after H_2_O_2_ and amyloid beta (A*β*) treatment, protecting against oxidative stress and apoptosis in PC12 and NT2 cells, respectively [77, 78]. Alternatively, PI-3K/Akt activation may promote neuronal and glial antioxidant defences by stimulation of the Nrf-2/antioxidant responsive element (ARE) [79].

Another target for insulin-induced PI-3K/Akt pathway is CREB, which, upon phosphorylation at Ser133, enhances mitochondrial membrane potential (ΔΨ_*m*_), intracellular ATP levels, NAD(P)H redox state, and hexokinase activity, that is, improves adult neuronal glucose metabolism and axonal outgrowth [80]. Interestingly, this Akt-mediated modulation of neuronal mitochondrial function and caspase activity has been correlated with changes in Bcl-2 expression (and its interaction with Bad) and/or with increased glucose transporter-1 (GLUT1) expression (and stimulation of glycolysis and protection against mitochondrial permeability transition pore opening and cytochrome c release) [49, 80]. This may, at least partially, constitute an explanation for the apoptotic cell death that arises from the loss of neurotrophic support and subsequent depletion of PI3K/Akt signaling [80].

### 2.4. SHC/ERK1/2 Signaling Pathway

ERK1/2 forms a parallel branch to PI3K signaling. After phosphorylation of IR, the adapter protein SHC (Src homology-2 domain-containing) binds to IR and Grb2 is attracted to bind to SHC via its SH2 domains, thus activating the ERK1/2 signaling pathway. SH3 domains are other Grb2 domains that interact with guanine nucleotide exchange son of sevenless (SOS) protein, stimulating the exchange of GDP for GTP at Ras, which becomes active and then recruits the Ser/Thr kinase Raf. Subsequent activation of MEK (or MAP2K, mitogen-activated protein kinase kinase) leads to phosphorylation (and activation) of ERK1/2 on Thr and Tyr residues [52, 81–83], culminating in activation of several transcription factors that control gene expression (e.g., Ets-like protein-1 (Elk-1) and c-Myc) [46]. 

Interestingly, ERK1/2 activation was originally described to play an antiapoptotic role in neurons via phosphorylation of Bad at Ser112 [84, 85]. Conversely, others showed the involvement of active ERK1/2 in synaptic plasticity and cell death [86–89], especially after oxidative stress [90] and N-methyl *D*-aspartate (NMDA) receptor-mediated excitotoxicity [87].

Traditionally, insulin signaling through PI3K-dependent pathway was considered as functionally separated from ERK- or MAPK-dependent signals, with PI3K controlling metabolism, while the mitogenic role was related with ERK1/2 [91]. However, others proposed that some cross-talk may occur between both signaling pathways, with Raf being the possible link, at least in glutamate-induced oxidative stress [82]. This cross-talk may have an anti-apoptotic role through Bad phosphorylation at Ser136 [92]. Interestingly, Subramaniam et al. [88] showed that ERK1/2 could be inhibited by IGF-1 via PI3K-dependent Raf phosphorylation at Ser259, thus protecting cerebellar granule neurons from cell death. So this could constitute an alternative pathway (to PI3K/Akt-induced phosphorylation of Bad, caspases and FoxO3) to promote neuronal survival [46]. 

Besides PI3K/Akt and ERK1/2 signaling pathways, insulin has also been described to protect against neuronal and non-neuronal apoptosis via activation of MAPK signaling [93, 94] (namely, p38 MAPK) and suppression of caspase-3 activity [95]. This was further confirmed by other studies showing that c-Jun N-terminal kinase (JNK) downregulation promoted cell survival [96, 97]. Activation of p38 MAPK by insulin/IGF-1 was also shown to stimulate glucose transport [98–102] and antioxidant-related genes in nonneuronal cells submitted to oxidative stress [103]. Surprisingly, little or no effect on MAPK activation occurred upon insulin/IGF-1 administration in embryonic dorsal root ganglion or adult sensory neurons [80, 104].

## 3. Role of Insulin and IGF-1 in the Brain

### 3.1. Glucose Metabolism

Taken together, we can infer that the complexity of insulin/IGF-1-mediated IR/IGF-1R signaling pathways may play a crucial role in CNS, including regulation of brain metabolism [105–108], neuronal growth and differentiation [16, 105, 109], or neuromodulation [105, 110–112]. Interestingly, tissue (and cellular) dependence on insulin is such that its restriction under some damaging conditions results in cell atrophy and apoptotic death [80].

The most well-known insulin effect is the regulation of peripheral glucose transport and metabolism [2, 113, 114]. However, the precise underlying mechanisms were unravelled only in the last decade and appear to involve a major integration of nutritional and hormonal peripheral signals, mediated by specialized groups of neurons from the arcuate nucleus of the hypothalamus, the glucosensing neurons [115]. These neurons respond to peripheral signals required for the regulation of ingestive behaviour and energy homeostasis. Besides insulin, such signals also include the anorexigenic peptides proopiomelanocortin (POMC) and cocaine- and amphetamine-regulated transcript (CART), as well as the orexigenic neuropeptide Y (NPY) and agouti-related peptide (AgRP) [116]. Under supraphysiological glucose levels, brain insulin signaling activation results in hyperpolarization of glucosensing neurons, activating K^+^_ATP_ channels and reducing neuronal firing (probably due to the inactivation of POMC neurons), thus decreasing body weight [39, 45, 116]. Conversely, impairment of brain insulin signaling (as occurs in peripheral insulin resistance) activates JNK, phosphorylating IRS-1 at Ser/Thr and promoting a feedback inhibition of IR, thus resulting in increased body weight. This orexigenic effect is mediated by the activation of arcuate neurons containing NPY, AgRP, and GABA [39, 45, 117]. Additionally, besides controlling body weight, hypothalamic insulin also regulates hepatic glucose production, and, upon impairment of hypothalamic insulin signaling, the subsequent decrease in hepatic sensitivity to the circulating insulin stimulates hepatic glucose production [39, 118, 119]. This suggests that insulin elicits a CNS (hypothalamus)-liver axis response to regulate hepatic glucose production [118].

Although traditionally brain glucose metabolism was considered essentially insulin insensitive [120], some evidences led to the hypothesis that insulin may regulate glucose metabolism only in glia [33, 121, 122]. However, more recent studies suggested that cerebral glucose metabolism may be controlled by neuronal insulin/IR signaling pathways [2, 80, 123, 124]. This hypothesis is supported by the overlapping distributions of insulin, IR, and glucose transporters (GLUTs) isoforms 1 and 4 in selective brain regions (e.g., hippocampus and choroid plexus) [15]. Furthermore, changes in circulating insulin levels were described to modulate cerebellar GLUT4 expression [125], and hyperinsulinemia led to regional changes in glucose utilization in rodent brain [126]. But the most striking evidence for insulin-mediated brain glucose metabolism came from Bingham et al. [127], showing that fasting insulin levels stimulate global glucose metabolism maximally in human brain cortex, either directly (as in peripheral tissues) or indirectly (via insulin-stimulated neuronal activation). These authors also suggested that, if the recruitment of GLUTs to the plasma membrane and subsequent increment in glucose uptake was a direct effect of insulin, this might involve the partially insulin-sensitive glial GLUT1, since the main neuronal glucose transporter (GLUT3) is insulin insensitive.

An alternative pathway for insulin to provide energy for neurons involves inhibition of neuronal norepinephrine uptake, with subsequent activation of glial *β*-adrenoreceptors and glucose extrusion from glial glycogen stores, namely, in astrocytes [2, 120]. As a result, astrocytic glycogen can be converted to glucose, which is then transported to the extracellular fluid via insulin-stimulated GLUT1, constituting an additional energy source for neurons [2, 128].

Taken together, these evidences suggest that any deleterious interference in the cross-talk between insulin and neuronal glucose metabolism may impair ATP synthesis and culminate in neuronal apoptosis [129]. In this regard, Wu et al. [130] showed that insulin prevented serumdeprivation-induced apoptosis in R28 rat retinal neurons, while Koo and Vaziri [131] hypothesized that insulin could also prevent oxidation of glucose transporters or stimulate antioxidant defence mechanisms in type 1 diabetic streptozotocin (STZ) rats, thus stimulating intracellular metabolism. Additionally, we reported that insulin-induced IR/IGF-1R activation and subsequent PI3K/Akt signaling prevented the decrease in hexokinase-II expression, thus stimulating glycolysis upon oxidative stress in rat brain cultured neurons [64, 132]. This further supports the idea that brain is both an insulin- and glucose-sensitive tissue [133]. Conversely, other studies failed to show acute effects of insulin on glucose transport into the brain [134, 135].

### 3.2. Other Roles for Insulin in Brain: Synaptic Transmission and Memory/Learning

Some authors hypothesized that brain insulin may play other roles apart from glucose metabolism, based on the heterogenous distribution of IR in brain [15], the poor correlation between IR location and neuronal energy utilization, the insulin-independent neuronal glucose uptake, the neuromodulatory role of insulin in invertebrates, its action on neuronal norepinephrine and serotonin uptake, and its relation to NPY [2]. Among such roles, brain insulin has been proposed to increase neurite outgrowth [136, 137] and regeneration of small myelinated fibers [2, 138], maintain cortical, sympathetic and sensory neuronal survival during nervous system development [139, 140], stimulate neuronal protein synthesis [2], and improve synaptic activity and plasticity, memory formation, and storage [110], as well as neuroprotection [141–143]. In this regard, insulin administration has been shown to improve memory/learning in rats [39] and in healthy humans (after intranasal administration), without changes in peripheral glycemia [12, 144]. Systemic insulin infusion also improved verbal memory and attention [145].

Interestingly, these results appear to correlate with high IR expression in hypothalamus and limbic system (hippocampus, pyriform cortex, and amygdala) [12, 15, 115] and may be associated with the well-known effect of insulin on synaptic transmission (e.g., monoamines) [15, 146, 147]. At this respect, about 30 years ago, insulin was described to promote epinephrine and norepinephrine release in adrenergic terminals [148], inhibit synaptic reuptake of norepinephrine, modify catecholamine kinetics, and stimulate neuronal serotonin uptake [42, 149–152]. More recently, some authors reported that insulin modulated the expression of NMDA receptors, increasing neuronal Ca^2+^ influx and reinforcing synaptic communication between neurons [153], and also modulated long-term potentiation, a molecular model of learning [126, 154]. This was further supported by insulin-mediated control of cell surface glutamate and GABA receptor density (via the modulation of receptor targeting to the membrane and endocytic internalization), thus affecting synaptic plasticity [31]. Additionally, we showed that insulin prevented the decrease in GABA and glutamate uptake and their increased extrasynaptosomal levels in rat synaptosomes after oxidative stress and/or type 2 diabetes [155, 156]. Apparently, such neuromodulatory role of insulin could arise from its direct effect on neurotransmitter transport or from decreased ATP levels and subsequent reversal of the amino acid transporters [155, 156], thus protecting neurons against damaging effects of excitotoxicity or oxidative stress. 

### 3.3. The Neuroprotective Role of Insulin 

**Figure 1:**
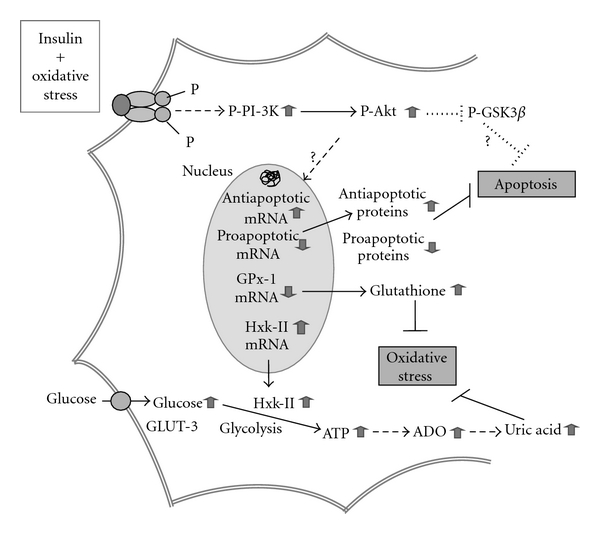
Stimulation of PI-3K/Akt signaling pathway after activation of IR/IGF-1R mediates insulin neuroprotection against damaging conditions. For example, insulin administration under oxidative stress phosphorylates IR and/or IGF-1R, which in turn activate the PI-3K/Akt signaling pathway, regulating the expression of “candidate” proteins, namely, glutathione peroxidase-1 (GPx-1), hexokinase-II (Hxk-II), and also the antiapoptotic Bcl-2 and the proapoptotic caspase-3. Thus, oxidative stress, impaired glucose metabolism and, neuronal apoptosis are counterbalanced. Insulin also interferes with GSK-3*β* signaling, decreasing its activated form and inhibiting apoptotic neuronal death under oxidized conditions.

Regarding this issue, we and others showed that insulin or IGF-1 attenuated both retinal and brain neuronal apoptosis induced by damaging conditions (e.g., oxidative stress) [64, 130, 157–162]. Apparently, such neuroprotection could arise from restoration of IR/IGF-1R signaling-mediated gene transcription (e.g., increased hexokinase-II and Bcl-2 and decreased glutathione peroxidase and caspase-3 expression) [64], thus improving neuronal glucose metabolism [1, 25, 132, 163, 164] and antioxidant defences [157] (Figure 1). Others proposed an alternative antiapoptotic mechanism involving IGF-1-induced activation of IGF-1R/PI-3K/Akt signaling and subsequent prevention of caspase activation via phosphorylation of the survival transcription factor CREB (activated), GSK-3*β* (proapoptotic), and transcription factor of the Forkhead box 1 (FoxO1, inactivated) [104, 165]. Insulin-induced antiapoptotic effect might also arise from neuronal SAPK inhibition [166]. Surprisingly, Schubert et al. [167] described that, in NIRKO mice (knocked out for neuronal IR), insulin treatment was capable to circumvent neuronal apoptosis in an IR-dependent manner. In contrast, Ryu et al. [160] failed to show protection by IGF-1 against excitotoxic or oxidative stress-induced necrosis, despite a decrement in neuronal apoptosis. Additionally, insulin induced free radical generation and lipid oxidation (leading to neuronal necrosis), in a protein-kinase-C (PKC-) dependent process [160, 168].

Independently of the underlying signaling pathway, insulin has been increasingly shown to play a neuroprotective role against several damaging conditions, including oxidative stress, and to mitigate neuronal apoptotic death [1, 157]. Since some of these injuries may constitute the underlying mechanisms of brain dysfunction associated with several pathologies (e.g., diabetes, aging and age-related diseases, as Alzheimer's disease (AD)), next, we will discuss the effect of insulin under such pathologies.

## 4. Protection by Insulin against Diabetes and Its Long-Term Complications in CNS

Diabetes mellitus, one of the most common metabolic disorders, is a major disorder of insulin regulation. Diabetes has reached epidemic proportions in western countries, and, according to World Health Organization estimates, it will affect ~300 million people worldwide in 2025, rendering diabetes an important public health concern in 21st century [169]. This is further aggravated by the increasing prevalence of diabetes with aging, risk factors associated with modern lifestyle in developed and under development countries (e.g., higher life expectancy, obesity, sedentarism, hypertension, hyperlipidemia, and genetic factors) [170], and its severe long-term complications [171] (e.g., cardiovascular disease, renal failure, retinopathy, stroke, and peripheral and autonomic neuropathy) [84, 172].

A consequence of diabetes, mostly of type 2 diabetes, is insulin resistance and chronic peripheral hyperinsulinemia, accompanied by downregulated insulin transport into the brain and its subsequent deprivation from insulin beneficial effects [173]. Indeed, diabetes-associated disruption between insulin activity and glucose metabolism results in decreased cerebral blood flow and oxidative glucose metabolism [46, 174, 175] (which may also arise from the impaired blood-brain glucose transport) [176]. This hypothesis is supported by the downregulation of neuronal insulin signaling pathways and brain glycolytic enzymes in uncontrolled diabetes [25, 176] (probably due to the inhibition of IR phosphorylation) that may culminate in progressive impairment in learning, memory, and cognition [25]. However, Seaquist et al. [134] failed to show any effect of short-term hyperglycemia in glucose transport. 

In previous studies, we described that, despite the apparent protection against oxidative stress in type 2 diabetic Goto-Kakizaki (GK) rat brain synaptosomes (probably due to higher plasma and brain vitamin E levels) [177], they had lower synaptosomal membrane potential and ATP/ADP levels [155]. These results were (at least partially) explained by the diabetes-related impairment of brain mitochondrial electron transfer chain, which might be exacerbated by aging and/or amyloid *β* peptide (A*β*) [178], and were counteracted by the antioxidant coenzyme CoQ10 [179] or insulin [180]. This reinforces the involvement of oxidative stress and/or metabolic/mitochondrial dysfunction in long-term damaging effects of diabetes [178–181].

Although the underlying mechanisms of diabetes-induced mitochondrial dysfunction are not completely understood, it has been proposed that increased mitochondrial nitric oxide synthase (NOS) activity (and subsequent nitric oxide (NO^•^) production) may inhibit mitochondrial complexes III and IV and ATP synthase, either by nitrosylation or protein thiol oxidation [182]. This may lead to impairment of ATP production, decreased ΔΨ_*m*_, opening of mitochondrial permeability transition pore (mPTP), and cell death [182]. 

Another consequence of diabetes is oxidative stress, which may arise from several mechanisms related with chronic hyperglycemia: advanced glycation endproducts (AGEs) formation, glucose autoxidation, mitochondrial dysfunction, endoplasmic reticulum stress, and impaired antioxidant defences [183, 184], culminating in caspase-dependent neuronal death [171, 183]. Indeed, dorsal root ganglion neurons from diabetic rats displayed increased apoptotic markers (e.g., cleaved caspase-3, positive TUNEL staining [171], and PARP-1 activation [185]). Interestingly, Li et al. [183] observed that hippocampal CA1 and CA2 neuronal density from type 1 diabetic rats was lower than in type 2 diabetic animals, whilst specific markers of apoptosis (e.g., TUNEL staining, Fas and Bax expression, cytosolic AIF, expression and activity of caspases-3 and -12) and nuclear labeling of 8-hydroxy-2′-deoxyguanosine (8-OHdG) were higher, suggesting that oxidative stress could be the link to apoptotic activity in type 1 diabetes. 

Besides brain glucose metabolism and oxidative stress, diabetes may also impair neurotransmission. Indeed, glutamate affinity for AMPA, but not NMDA receptors, was shown to be decreased in type 1 diabetic STZ rats [172]. Others reported an increase in taurine and GABA transport in diabetic rat retina and retinal pigment epithelium [111] and that hyperglycemia impaired glutamate release upon ischemia/reperfusion in both nondiabetic [186] and diabetic animals [187, 188]. In this regard, we also showed that GABA levels were decreased in STZ-diabetic synaptosomes compared to control synaptosomes [189] and that insulin could modulate rat brain synaptosomal GABA and/or glutamate transport under oxidation and/or type 2 diabetes [155, 156].

As an antidiabetes therapy, insulin has been the best studied and more efficient pharmacological compound mainly used in the treatment of type 1 diabetes. Nevertheless, it has been also increasingly used in the treatment of type 2 diabetes noncontrolled by diet, exercise or, oral antidiabetic agents, and its use has been shown to decrease microvascular complications and mortality [190, 191]. In this perspective, it has been increasingly suggested that all diabetic patients could be insulin treated, independently of disease progression [191]. In terms of subcellular effects of insulin on diabetes, Guyot et al. [186] reported that the insulin-induced increase in extraneuronal glutamate in type 1 diabetic STZ rats submitted to ischemia could arise from stimulation of astrocytic glucose uptake (instead of neuronal glucose uptake) with subsequent decrement in neuronal glucose metabolism and in the energy available for neurotransmitter uptake. This effect on neurotransmission was even more pronounced in STZ rats given a high dose of insulin, resulting in a prolonged release of aspartate, glutamate, taurine, and GABA [186]. Noteworthy, the extracellular increase in GABA as well as in the number of GABA_A_ receptors upon insulin treatment might protect against the cytotoxic effects of released excitatory amino acids. Importantly, the damaging effects of diabetes on neurotransmission may be potentiated by impairment of Ca^2+^ uptake to the endoplasmic reticulum and/or mitochondria, further exacerbating neuronal excitotoxicity [172]. 

From the abovementioned, it seems that only diabetes-related hyperglycemia account for the long-term damage and that, by normalizing blood glucose levels, insulin might protect against those injuries. Nevertheless, it has been also increasingly demonstrated that recurrent hypoglycemic episodes (the most common side-effect of insulin therapy) may adversely affect diabetic CNS [84, 172], resulting in motor incapacity, seizures, or cognitive damage, mainly in aged patients [192]. At this respect, it has been shown that recurrent hypoglycemia affected the endogenous levels of metabolites indirectly linked with brain glucose metabolism (e.g., glutamate, leading to excitotoxicity), accelerated lipolysis (increasing the formation of highly oxidizable polyunsatured fatty acids), impaired protein synthesis, ion homeostasis, and mitochondrial function, culminating in neuronal dysfunction [120, 193]. In a recent study, our group showed that, although insulin was able to protect type 1 diabetic STZ rats (either exposed or not to A*β*) against brain oxidative stress and mitochondrial dysfunction [180, 194], this was not the case in STZ rat brain after an acute hypoglycemia episode [28]. This suggested that poor glycemic control might damage brain areas involved in learning and memory [28], thus limiting the analysis of insulin and hyperglycemia roles in diabetic brain [11]. Under this perspective, it is not surprising that brain requires robust neuroendocrine counterregulatory mechanisms to maintain blood glucose within a narrow, nondeleterious range. 

As described before, diabetes has been widely associated with slowly progressive end-organ damage in brain [27, 195], resulting in diabetic neuropathy and/or mild to moderately impaired cognitive function, both in type 1 and type 2 diabetic patients [11]. However, clinically relevant deficits mostly occur in elderly type 2 diabetic patients, probably due to a complex interaction between diabetes and the normal brain aging [11]. Although the molecular mechanisms underlying such long-term effects of diabetes in CNS remain unclear, it has been hypothesized the involvement of AGEs formation, aldose reductase activity, oxidative stress, activation of protein kinase C, and increased hexosamine pathway flux [91, 171, 193, 196, 197]. In a very recent study, Soeda et al. [198] described an increase in insulin signaling upon neuronal mutation of SH2-containing inositol 5′-phosphatase 2 (SHIP2, a negative regulator of phosphatidylinositol 3, 4, 5-trisphosphate-mediated signals) and that SHIP2 levels were also increased in type 2 diabetes *db/db* mice brain. Surprisingly, SHIP2 inhibition ameliorated hippocampal synaptic plasticity and memory formation. Conversely, when SHIP2 was overexpressed in mice, Akt-mediated IR/IGF-1R signaling was impaired, the neuroprotection by insulin/IGF-1 was attenuated, and increased neuronal apoptosis and impaired memory were also reported [198]. 

Interestingly, insulin treatment has been described to prevent biochemical and pathological indices of peripheral sensory neuropathy in animal models of type 1 diabetes at doses that did not impact on hyperglycemia [80], suggesting that insulin therapy might not only ameliorate peripheral diabetic complications but might also improve brain function in diabetic patients [199–201]. Furthermore, this points to the existence of a link between insulin, cognitive decline and dementia, and type 2 diabetes [27, 195]. 

## 5. Insulin in Aging and Longevity

Aging can be defined as a time-dependent loss of fitness that begins after the organism attains its maximum reproductive competence [202], that is, an increased susceptibility to stress [203] that culminates in an increased incidence of chronic diseases and an exponential increase in the chance of dying [202]. Although the molecular mechanisms underlying aging remain controversial, Harman proposed, in 1968, that oxidized macromolecules accumulate with age, decreasing cell function and shortening lifespan—the Free Radical Theory of Aging [204]. If this was as simple as Harman proposed, antioxidant therapy would prevent aging-related damage to tissues. However, nutritional and genetic studies aiming to increase lifespan by boosting antioxidant defences were mostly unsuccessful [205]. Indeed, supplementation with vitamins E or C was not able to reduce mortality in several clinical trials [206, 207]. Moreover, studies involving overexpression of antioxidant enzymes (e.g., superoxide dismutase and/or catalase) in mice failed to increase lifespan, despite the decrease in oxidized macromolecules [208]. Conversely, overexpression of the peroxidase and redox-active thioredoxin 1 [209] and mitochondrial-targeted catalase [210] were shown to prolong mouse lifespan. 

A parallel antiaging paradigm has been increasingly proposed, in which the effect of genetic changes or caloric restriction in insulin signaling pathway might slow the rate of living, thus decreasing metabolism and oxidative stress [211–214]. This hypothesis has been supported by studies showing that caloric or dietary restriction inhibited insulin signaling cascades that regulate glucose intake, prolonging rodent longevity [215]. However, this does not explain why the decrease in ROS levels occurs, despite the increased metabolic rate observed under such conditions [216]. 

More recently, it has been suggested that epigenetic factors modulated by aging (e.g., histone deacetylase family, namely, sirtuins, histone acetylases and DNA methyltransferases) impose a metabolic (redox) shift towards an increased reliance on glycolysis (instead of mitochondrial metabolism), probably mediated by age- and sedentarism-related insulin resistance (conditions that require lower metabolic demands). Accordingly, oxidized Kelch-like ECH-associated protein 1 (Keap-1) may block NF-E2-related factor (Nrf) release to signal mitochondrial biogenesis, thus decreasing mitochondrial synthesis and forcing cells to rely mostly on glycolysis [205]. As a consequence, aged tissue becomes increasingly unable to cope with energy demands or stress, initiating a catastrophic cycle of oxidized membrane receptors, signaling molecules, transcription factors and epigenetic transcriptional regulators, culminating in cell death and organ failure [205]. Although the mechanisms remain unknown, it is also believed that, in this age-related oxidized environment, IR/IGF-1R may be constitutively oxidized (by free radicals and/or AGEs, which are also increased in aging [217]). This process may be due to the failure of oxidized protein tyrosine phosphatase-1b (PTP1b) to reactivate the receptor, thus limiting subsequent insulin signaling progression and impairing translocation of glucose transporters into the membrane. As a consequence, glucose levels may rise, and, although insulin production may also be stimulated, it may have no effect—the well-known insulin resistance condition that occurs in type 2 diabetes and aging [1, 205]. Insulin resistance further potentiates the formation of AGEs, creating a damaging vicious cycle on aging brain [218].

Age-associated changes in IR signaling may also arise from increased cholesterol levels and decreased membrane fluidity (impairing lateral movements of lipid rafts) or from modification of IR internalization, reexpression, or degradation by the proteasome [1]. Noteworthy, aging has also been associated with a decrease in brain IR number and binding capacity, namely, in hippocampus, cortex, and choroid plexus [31, 219, 220].

Undissociated from aging is the longevity, which can be defined as the property of approaching the species-specific maximum lifespan, that is, the oldest observed age of death in the species [202]. Rather than environmental conditions, longevity depends mostly on genetics [202]. Increasing evidence points towards the idea that decreased insulin signaling pathway may promote longevity in several species, including yeast, worms, *Drosophila*, mice, and man [212, 221–225]. A hypothesis further supported by increased adiponectin and peroxisome proliferator-activated receptor *γ*-2 (PPAR*γ*-2) gene (two well-known insulin sensitizers) in centenarians and long-lived men, whose type 2 diabetes incidence was also dramatically decreased [226]. Additionally, single mutation of daf-2 (an IR/IGF-1R homolog) resulted in an increase in lifespan, via the nuclear translocation of the FoxO family transcription factor DAF-16 [227]. In this regard, evidences suggested that 14-3-3 molecules may complex with sirtuin 2 (Sir2) upon stress, activating nuclear FoxO/DAF-16, while the inactive FoxO/DAF-16 remained in cytoplasm upon normal insulin signaling [228]. Null mutations in *Drosophila* genes that encode IR or IRS homologues were shown to extend lifespan of female fruit flies [202]. Similarly, overexpression of IRS-1 in mice was shown to decrease lifespan [223], and overexpression of IRS-1, 2, 3, or 4 in adipose cells was shown to stimulate GLUT4 translocation independently from insulin [229]. Accordingly, mice lacking IRS-2 had extended lifespan [222]. Surprisingly, IR overexpression in mammary epithelial cells resulted in a tumorigenic phenotype [230], suggesting that its continuous activation upon age-related oxidative stress might not be beneficial. Recent studies suggest that reversible control of cGMP via phosphodiesterase regulation might modulate insulin production and, thus, could constitute a key regulatory messenger of lifespan extension [227].

Studies in centenarian people and caloric restriction in rodents and nonhuman primates suggested that prerequisites for longevity include (besides the above-mentioned increased insulin sensitivity and subsequent normal IR signal transduction) decreased fasting glucose and oxidative stress [1, 231]. In line with this, decreased mitochondrial function has been suggested to, in some circumstances, increase lifespan [232]. This idea is supported by studies in the *clk-1* mutant worms, showing reduced respiratory, developmental and behavioural rates, but longer lifespan. Such phenomena may be related with the fact that CLK-1 is essential for ubiquinone synthesis, an important component of the mitochondrial electron respiratory chain [232]. Similar results have been described in *Drosophila* mutants with lower expression of electron transfer chain components in adult neurons [233] and in a mouse model with decreased activity of cytochrome c oxidase complex [234]. 

In this regard, type 2 diabetes appears to be, at least partially, a model for premature aging. This idea has been further confirmed by a decrease of cellular replicative senescence in diabetic subjects [1]. Thus, one of the main challenges for the next decades will be to unravel the complex interactions between aging and diabetes that result in insulin resistance, allowing the development of more efficient preventive and therapeutic strategies to overcome age-related diseases (e.g., Alzheimer's disease (AD)).

Taken together, if (1) aging is frequently related with sedentarism and (2) the organism does not require extra demands for energy, then it can survive longer on the lower energy levels resulting from glycolysis. This can occur by downregulating the mitochondrial electron transport chain components and the activities of several redox-sensitive transcription factors, enzymes, transporters, and signaling proteins (e.g., IR), as has been widely described [205]. However, the challenge becomes when the aged organism faces a stress condition.

## 6. Insulin in an Age-Related Neurodegenerative Disorder: Alzheimer's Disease

AD is a complex and common neurodegenerative disease that afflicted 26.6 million people worldwide in 2006, a number that can quadruplicate by 2050 [235]. Clinically, this disease has an insidious onset, typically beginning with a subtle decline in memory that progresses to global deterioration in cognitive and adaptive function [11]. Neuropathologically, is characterized by the presence of extracellular senile plaques (SP), intracellular neurofibrillary tangles (NFT), and loss of basal forebrain cholinergic neurons that innervate the hippocampus and cortex [236]. While NFTs are formed from paired helical filaments composed of neurofilaments and hyperphosphorylated tau protein, SP arises mostly after deposition of amyloid *β* (A*β*; a 39–43 amino acid peptide derived from the proteolytic cleavage of a larger amyloid *β* precursor protein (A*β*PP) by the *β*- and *γ*-secretases) [237]. The most common resulting fragments are either 40 or 42 amino acids in length (A*β*_1−40_ and A*β*_1−42_) [11]. Although the initiating factor(s) for AD remain unclear, some authors suggested that they might involve intracellular (rather than extracellular) accumulation of A*β* [39], leading to neurite dystrophy and degeneration, postsynaptic protein loss, and eventual death of neocortical, hippocampal, and subcortical neurons [39, 44, 179]. Importantly, A*β* accumulation is also a common process in normal aged human, but its massive deposition in AD [238] may result from either an overproduction of A*β*PP, impaired A*β*PP proteolytic processing, and/or mutations in the genes encoding for A*β*PP or presenilins [179].

Only 5% (or less) of all AD cases have an early onset, autosomal dominant familial origin, probably due to missense mutations in presenilin (PS) genes 1 or 2 in chromosomes 14 and 1, or in the A*β*PP gene in chromosome 21 [239]. Additionally, allelic abnormalities of the apolipoprotein E (APOE) gene on chromosome 19 are related with early onset and increased severity of inherited and sporadic AD [239]. On the contrary, most of AD cases (95% or more) are sporadic in origin, aging being the main risk factor [11, 232]. Although sporadic AD is faced as a “disease” of aging, this does not necessarily imply that aging *per se* is a disease, and we must bear in mind that the boundaries between late-onset AD and “normal” aging are not absolute. This may be due to (1) increasing evidences from epidemiological, immunohistochemical, and molecular genetics studies suggesting a heterogenous etiology for AD [240] and (2) the fact that an unquestionable AD diagnosis is only possible after the patient's death, through *post mortem *morphological and histological brain analysis [179]. Indeed, a decline in neuropsychologic test performance, brain atrophy, neuronal loss, and plaque/tangle deposition also occur with aging in the absence of dementia [241]. Therefore, it is reasonable to consider the causal molecular events for sporadic AD within the aging spectrum, rather than distinct disease phenomena. 

Although the exact pathophysiologic mechanisms underlying signal transduction abnormalities and neurodegeneration in AD brain remain unknown, oxidative stress and metabolic dysfunction appear to be involved [68, 242, 243]. Indeed, A*β* was shown to directly induce overproduction of reactive oxygen and nitrogen species and neurotoxicity [244]. This was further exacerbated by A*β*-associated decreased plasma antioxidant defences (e.g., uric acid, glutathione, catalase, superoxide dismutase, glutathione peroxidise, and, reductase), as described by several authors [245–248]. Additionally, antioxidant supplementation (e.g., vitamin E plus vitamin C, and donezepil, an acetylcholinesterase inhibitor, plus vitamin E) was shown to have beneficial effects in AD [249, 250]. Similarly, vitamin E, idebenone, uric acid, or glutathione prevented A*β* neurotoxicity in human and rat cortical and hippocampal neurons and delayed disease progression in AD patients [251]. Moreover, an induction of apolipoprotein D expression (a protein that appears to function as lipid antioxidant and to extend lifespan in *Drosophila*) has been suggested to constitute an age-related stress-resistance mechanism in AD brains [232]. 

Taken together, these data unequivocally demonstrate the involvement of oxidative stress in AD and suggest a potential therapeutic role for antioxidant supplementation [245, 252]. However, others studies failed to show any protection induced by antioxidant treatment in AD [249, 253]. Although indirectly, the involvement of oxidative stress in AD pathophysiology was further reinforced by the observation of AD neuropil enrichment in AGEs and redox active metal ions (e.g., zinc, iron), particularly in SP, and NFT [179, 251] (as detected in brain cortex, hippocampus, and basal nucleus of Meynert from AD patients) [254]. To further increase the complexity on this subject, oxidative stress in AD may also arise from mitochondrial dysfunction, involving decreased activity of mitochondrial complex IV and decreased ATP/ADP, as revised by Moreira et al. [11]. In fact, evidences widely implicate metabolic defects in AD, the lower brain metabolic rate being one of the best-documented abnormalities that occurs early in the pathology [255]. This may involve impaired glucose uptake and metabolism and also a slightly decreased cerebral metabolic rate of oxygen (at the beginning of AD) [256, 257], preceding any evidence for functional impairment by neuropsychological testing or brain atrophy neuroimaging [258]. Thus, it is believed that impaired AD brain metabolism may be a cause, rather than a consequence, of neurodegeneration [259].

Although the underlying causes of reduced metabolism in AD are not completely understood, it has been reported that atrophy of cerebral vasculature (the major metabolic exchange surface of brain), a decrement in brain glucose transport activity, or even impaired insulin signaling might play a crucial role [11]. Indeed, Liu et al. [260] proposed that GLUT1 and GLUT3 could be downregulated in AD, thus impairing brain glucose uptake/metabolism. Additionally, several studies (including ours) showed that levels or activity of enzymes from intermediary metabolism (e.g., aconitase, glutamine synthetase, creatine kinase, pyruvate dehydrogenase, and *α*-ketoglutarate dehydrogenase) were decreased in AD brains and cells exposed to A*β* [261–264]. More specifically, Bubber et al. [265] observed that changes in tricarboxylic acid cycle enzymes' activities (mainly of the pyruvate dehydrogenase complex) correlated with the clinical state, suggesting a coordinated mitochondrial alteration in AD.

One of the most common changes in electron transport chain underlying mitochondrial dysfunction in AD is decreased cytochrome oxidase activity, described in such distinct AD human tissues as platelets [266, 267] and *post mortem* brain tissue [268, 269]. Furthermore, studies with cybrid cells demonstrated that AD platelet deficits in cytochrome oxidase could be transferred to cells depleted of mitochondrial DNA (mtDNA) (Rho0 cells), suggesting that mtDNA-associated mitochondrial dysfunction might play a role in AD neurodegeneration [266, 270, 271]. Valla and collaborators [272] also reported significant declines in complexes III and IV in AD lymphocyte mitochondria and a significant decline in complex IV in mild cognitive impairment lymphocyte mitochondria, suggesting that mitochondrial abnormalities could be present at the earliest symptomatic stages of the disease. Finally, enhanced mitochondria degradation was described in AD, leaving behind lysosomal detritus containing non-functional mitochondrial components [261, 273]. 

Noteworthy, among dementia-type disorders, AD has been increasingly associated with type 2 diabetes [27, 274, 275]. Indeed, two epidemiological studies (the Honolulu-Asia Aging Study, and the Rotterdam and Mayo studies) reported that type 2 diabetes increases the risk for AD, dependently on vascular dementia [175, 276, 277]. Furthermore, both pathologies share several common aspects, including aging-related processes, degeneration, high cholesterol levels, peripheral and CNS insulin resistance, dysfunctional IR and IR-mediated signaling, and decreased glucose transport and metabolism, despite the higher nonmetabolized glucose levels in cerebral blood [2, 5, 25, 27, 175, 256, 259, 275]. This led to the hypothesis that type-2-diabetes-mediated recurrent hyperinsulinemia/hypoglycemia episodes culminate in long-term changes in brain vasculature, neurodegeneration, and cognitive impairment, facilitating AD onset [30, 126, 275]. The existence of a correlation between the severity of neuritic plaques, NFT and cerebral amyloid angiopathy, and the presence of type 2 diabetes and apolipoprotein *ε*_4_ (APO*ε*_4_) allele further supports this hypothesis [25, 27, 278]. On the other hand, AD patients exhibited a higher risk for developing type 2 diabetes [279], and this led to the idea that brain IR signaling might be the missing link between brain neuronal loss and pancreatic *β*-cell loss in both diseases [27, 279].

More interestingly, a recent proposal refers that AD can be an “insulin-resistant brain state” [11], or even a “type 3 diabetes” [12, 280–283]. This can be supported by the reports on age- and AD-related decrease in insulin mRNA and protein levels [11, 282, 283], IR or IGF-1R expression [11, 40, 219, 284], IRS-1 and IRS-2 levels [11], markers of Tyr kinase activity (namely, active IRS-1, PI3K, and ERK1/2) [40, 175], or a reduced association of Shc with Grb2 [30]. Additionally, soluble A*β* oligomers were shown to interfere with IR function, probably due to a loss of these receptors at dendrites and their increased expression in the cell soma [285], despite no changes in absolute hippocampal neuronal IR levels [286]. Accordingly, Moloney et al. [40] suggested that damaged IR-associated neurotrophic and metabolic brain functions in AD neurons might arise after persistent and pathological hyperactivation of the Akt-mTOR-S6K signaling pathway, increasing IRS-1 phosphorylation at Ser312 or 616, culminating in IRS-1/2 degradation.

Concerning increased fasting plasma insulin, decreased CSF insulin levels, and/or decreased CSF/plasma insulin ratio in AD patients [11, 175], they are suggestive of insulin clearance impairment, which may elevate plasma A*β* levels [27, 274], due to the role of insulin in modulation of amyloid processing both in vivo and in vitro. In fact, brain insulin/IGF-1/Akt-mediated phosphorylation/inactivation of GSK-3*β* inhibited A*β* production [274, 287] and its abnormal intracellular accumulation, probably by increasing its extracellular secretion and by accelerating its trafficking from Golgi and trans-Golgi network to the plasma membrane [39, 40]. However, under insulin resistance conditions, despite the chronic peripheral hyperinsulinemia, downregulation of brain insulin synthesis and/or transport decreased brain insulin levels [218] and its subsequent signaling cascades, culminating in increased A*β* levels, as in Tg2576 AD transgenic mice [288]. More recently, Freude et al. [289] reported that IRS-2 deficiency in this mouse model of AD decreased both A*β*PP cleavage and A*β* levels in brain. Similar results were obtained upon selective neuronal disruption of IGF-1R in Tg2576 mice [289]. Alternatively, insulin/IGF-1 prevented A*β* accumulation by promoting its transport into CNS via A*β*-binding carrier proteins (e.g., transthyretin and albumin) [27, 200, 238, 290–292], or by insulin interfering with extracellular proteolytic A*β* degradation via insulin-degrading enzyme (IDE). This metalloprotease, that also catabolizes insulin and IGF-1, can be competitively inhibited by insulin resistance [30, 46, 126, 200, 218, 238, 278], impairing A*β* degradation, increasing its neurotoxicity and promoting AD [11, 46, 278]. Noteworthy, since brain IR does not desensitize, IDE may also constitute a negative feedback loop to control insulin action [31, 46], that is, PI3K/Akt activation by insulin may upregulate IDE, which may stop subsequent signaling and promote A*β* clearance in hippocampal neurons [31]. 

Insulin and IGF-1 were also described to modulate both physiological and abnormal neuronal tau protein phosphorylation, in a process involving Akt, GSK-3*β*, ERK1/2, and Cdk-5 [12, 25, 27, 30, 39, 40, 44]. Similarly, Sui et al. [57] observed that GSK-3*β* inhibition blocked specific phosphorylation of tau protein in PC12 cells. Moreover, in diabetic animals treated with insulin, a complete prevention of tau protein hyperphosphorylation was reported, probably resulting from reestablishment of brain insulin signaling [293]. This is further supported by the age-related tau protein hyperphosphorylation and CNS accumulation in both transgenic NIRKO mice (mice lacking brain/neuronal IR) [59] and IRS-2-deficient mice (in which brain IR is dysregulated) [294]. Interestingly, hyperinsulinemia in NIRKO mice [12, 167] and insulin treatment in human NT2 neurons [295] were described to decrease tau protein phosphorylation. Conversely, several authors described that in human SH-SY5Y neuroblastoma cells [59, 296] and rat primary cortical neurons [297], insulin exposure increased hyperphosphorylated tau protein levels, which was not transported into axons, thus accumulating and aggregating into NFTs in neuronal perikarya, and thereby promoting oxidative stress, apoptotic or necrotic death, and mitochondrial dysfunction associated with AD [30]. 

## 7. Is There a Therapeutic Window for Insulin against Diabetes- and Age-Related Neurodegeneration?

From all the above-mentioned evidences, we believe that a valuable therapeutic window for insulin against diabetes- and age-related neurodegenerative disorders (e.g., AD) may exist. Under these conditions, an inadequate trophic support to brain may occur due to lower insulin/IGF-1 levels and/or damaged IR/IGF-1R-mediated signaling, affecting gene transcription and culminating in neurodegeneration/death, cognitive dysfunction, and, ultimately, in long-term complications (in case of diabetes) and/or in AD (and other dementia) [46]. Thus, those symptoms should be alleviated upon increased insulin levels, accomplished by exogenous insulin plus glucose administration (to maintain euglycemia, avoiding the deleterious effects of hypoglycemia on memory and cognition) [30, 164, 175]. Interestingly, intranasal insulin administration has been increasingly considered as a potential peripheral therapy, with the advantage of penetrating the CNS within minutes (without affecting plasma glucose or insulin levels) [298]. This can occur via extracellular bulk flow transport along olfactory and trigeminal perivascular channels, but also through more traditional axonal transport pathways, culminating in memory improvement [39, 144, 298–300]. In this regard, a very recent clinical trial from Craft et al. [301] showed that treatment of AD or mildly cognitively impaired adults with intranasal insulin stabilized or improved cognition and cerebral glucose metabolism. Similarly, direct intracerebroventricular insulin administration was shown to improve memory performance (without changes in blood insulin or glucose concentrations), but this approach poses some questions concerning its human applicability [175]. Although the use of stem cells to deliver insulin or IGF-1 into brain has been considered as a hypothetical beneficial therapy (by increasing neuronal survival and decreasing oxidative stress in the CNS), safety and efficacy issues must first be improved [30].

However, chronically high insulin levels in brain can be deleterious [46], due to (1) desensitization of PI3K pathway and inadequate responses to other trophic factors, (2) potentiation of NMDA receptors and resultant excitotoxicity, and/or (3) competition with A*β* for IDE, increasing extracellular A*β* accumulation (meaning that excessive insulin must be removed to alleviate the competitive blockade of IDE) [44, 46]. A therapeutical alternative could be the use of antidiabetic insulin sensitizers (e.g., thiazolidinediones) that, under hyperinsulinemia, would decrease insulin availability to the brain without affecting glycemia [46]. Unfortunately, clinical trials showed that rosiglitazone, a thiazolidinedione, decreased cognitive performance in AD patients [302, 303]. Other therapeutical approaches could be (1) the small molecule insulin mimetic demethylasterriquinone B_1_ (DAQB_1_) that does not competitively inhibit IDE and modulates IR [304], (2) the delivery of insulin antibodies, or (3) insulin-inhibiting peptides into the brain [46]. Increased CNS IDE levels (via gene therapy or IDE infusion) would be also of therapeutical interest but is less practical. Given that activated PI3K/Akt inhibits GSK-3*β* and subsequent A*β* production and tau protein hyperphosphorylation (as well as increased A*β* clearance via stimulation of transthyretin and IDE), another strategy could be the improvement of insulin signaling [44, 46, 218]. In line with this, the use of GSK-3*β* inhibitors could be attractive, but it might also impair several vital physiological targets of this kinase or have no impact on other critical components of the neurodegenerative cascade [30, 218].

## 8. Conclusion

In the last three decades, brain insulin signaling has faced a novel and increased interest in neuroscience research, either in its signaling pathways and/or as a promising therapy against diabetes and age-related neurodegenerative disorders (e.g., AD). From the first studies recognizing the abundance of insulin and IR in brain to its potential involvement in numerous neurodegenerative diseases associated with diabetes (particularly type 2 diabetes) and aging mediated less than 20 years. Indeed, in 1999, Halter described that elderly people have impaired insulin sensitivity, which might account for by the slight age-related increase in fasting glucose levels and the delay in return to normal glucose levels after an oral glucose tolerance test. However, more than 10 years later, the elucidation on whether the origin of this insulin resistant state relies on aging *per se* or on external, lifestyle factors remains a matter of debate. In this regard, one of the main challenges for the next decades will be to unravel the complex interactions between aging and diabetes that underlie insulin resistance, allowing the development of more efficient preventive and therapeutic strategies to overcome age-related neurodegenerative diseases.

Concerning AD, a recent hypothesis points towards the idea that AD is the “brain-type diabetes.” This is supported by decreased number or binding capacity of brain IR in both AD patients and mouse models, increased risk for type 2 diabetes in AD patients (and vice versa), and the accumulation of hyperphosphorylated tau in the CNS of IRS-2-disrupted mice, a model of type 2 diabetes. Thus, restoring insulin levels and/or its receptor-mediated signaling cascades (without affecting blood glucose levels) constitute a potentially interesting therapeutic strategy against AD, due to the inhibition of A*β* production (and its increased clearance) and tau protein hyperphosphorylation, two well-known hallmarks of the pathology.

More recent studies focused on the importance of insulin/IR signaling in increased longevity. However, the results remain highly controversial. 

In summary, in CNS, rather than just an “acquired” peripheral hormone, insulin appears to be a naturally occurring peptide of the outmost importance.
